# Jebel Moya (Sudan): new dates from a mortuary complex at the southern Meroitic frontier

**DOI:** 10.1080/0067270X.2013.843258

**Published:** 2013-10-28

**Authors:** Michael Brass, Jean-Luc Schwenniger

**Affiliations:** a Institute of Archaeology, University College London, 31–34 Gordon Square, London, WC1H 0PY, United Kingdom; b Research Laboratory for Archaeology and the History of Art, University of Oxford, South Parks Road, Oxford, OX1 3QY, United Kingdom

**Keywords:** Jebel Moya, cemeteries, ceramics, Sudan, Meroe, OSL dating

## Abstract

This paper proposes a new chronology for the burial complex at Jebel Moya, south-central Sudan. It reassesses the body of evidence from Sir Henry Wellcome's original 1911–1914 excavations in order to place the site within a firm chronological framework by: (a) applying an attribute-based approach to discern discrete pottery assemblages; and (b) applying initial OSL dates to facilitate the reliable dating of this site for the first time. Jebel Moya is re-interpreted as a burial complex situated on the southern periphery of the late Meroitic state, and its potential to serve as a chronological and cultural reference point for future studies in south-central and southern Sudan is outlined.

## Abstract (FR)

Cet article propose une nouvelle chronologie pour Jebel Moya, site funéraire du centre-sud du Soudan. Les données provenant des fouilles qui y furent menées par Sir Henry Wellcome en 1911–14 sont réexaminées afin de placer le site dans une trame chronologique ferme, en employant une analyse par attributs pour définir des assemblages distincts de céramique, et en mettant en œuvre des datation OSL pour faciliter la première datation fiable du site. Nous réinterprétons Jebel Moya comme un site funéraire à la périphérie sud de l'état Méroitique tardif, et nous soulignons le potentiel du site pour servir de référence chronologique et culturelle à de futures études dans le sud et le centre-sud du Soudan.

## Background

**Figure 1.**
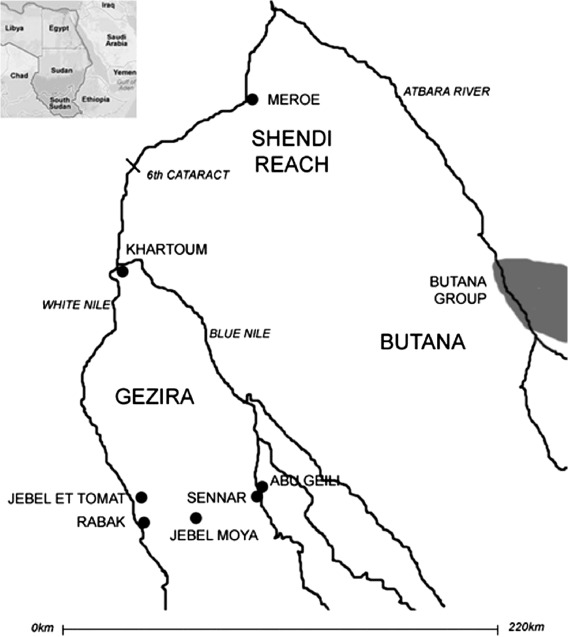
The location of Jebel Moya in south-central Sudan (adapted from Edwards (1989, Figure 1) and Winchell (2013, Figure 1.2)).

**Figure 2.**
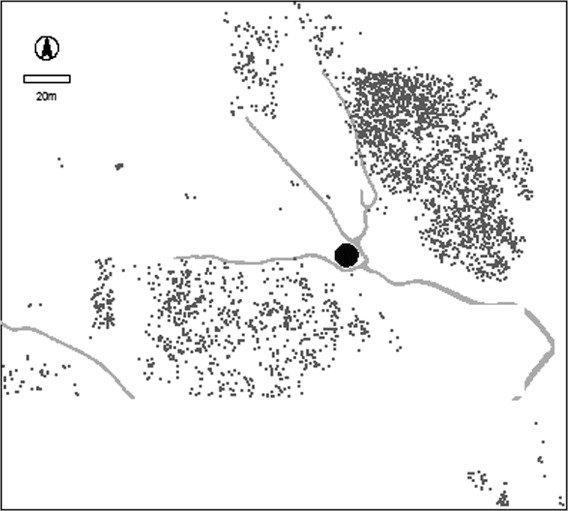
The distribution of burials in the Jebel Moya valley. The grey lines are water-eroded gullies and there is a large rock formation in the centre.

The Jebel Moya massif lies in the southern part of the Gezira Plain, Sudan, between the White and Blue Niles about 250 km south-southeast of Khartoum and approximately 310 km upstream from the Sixth Cataract (Figure 1). The area excavated at Jebel Moya is situated in a basin-like valley within the northeastern portion of the massif. Approximately a fifth of the basin's 10.4 ha was excavated over four seasons between January 1911 and April 1914 (Addison 1949), yielding 3135 human burials in 2791 graves, making it is the largest cemetery yet excavated in Northeast Africa (Figure 2). The precise dating of this site has long been in doubt and the aim of the present study is to define better the temporal context of this interpretively important assemblage.

The excavation of Jebel Moya was funded by Sir Henry Wellcome in the years leading up to the First World War, initially as research into a time period and area that interested him, but ultimately as a philanthropic gesture. Upon his death in 1936, the Wellcome Trust appointed Frank Addison to undertake an analysis of the archaeological materials, which had been shipped to England during the course of the excavations (Addison 1949). J.C. Trevor (Duckworth Laboratory, University of Cambridge) was commissioned to complete the osteological work; he brought in Ramkrishna Mukherjee and C. Radhakrishna Rao to undertake the majority of the analyses (Mukherjee *et al.* 1955). After the Second World War, the excavation records and osteological remains were deposited with the Duckworth Laboratory, where they remain. The majority of the remaining representative pottery assemblage was donated to the British Museum, with small pottery samples and most extant small finds going to the Petrie Museum (University College London), the Pitt Rivers Museum (University of Oxford) and the Museum of Archaeology and Anthropology (University of Cambridge) Finally, a few artefacts were ultimately returned to Sudan, while token collections of other artefactual materials were distributed to different museums outside the United Kingdom.

## Previous research: the Addison and Gerharz chronologies

Jebel Moya was initially dated by Addison (1949: 249–260) to *c*. 1000–400 BC. He later modified his dating to a period between the last centuries BC and the fourth century AD, which is roughly coeval with the Meroitic state to the north (Addison 1956). This radical change in dating, based upon the same pottery assemblages and the stratigraphic distribution of graves, is the primary reason why Jebel Moya's chronology has long been regarded as insecure. Gerharz (1994) has since revisited the issue, but drew his data from and based his conclusions solely on Addison's 1949 published Register of Graves. He did not re-examine the extant artefactual, ceramic, osteological or excavation records. Gerharz proposed three phases for the site based on the re-seriation of 465 grave inventories and radiocarbon dates from nearby sites that were claimed to possess similar types of artefacts. These may be summarised as follows:

### Gerharz's Phase I (5th millennium BC)

This was thought to be a period of sporadic occupations characterised by the ‘Dotted Wavy Line pottery tradition as identified from a small selection of the ceramic collection curated at the British Museum examined by Caneva (1991) and later verified by Manzo (1995).

### Gerharz's Phase II (3000-800 BC)

This is believed to have encompassed the majority of the graves across the valley. These graves comprise those burials that contain either no or very few associated grave goods. The chevron and cross-hatch decorated, thickened/everted rims attributed to this have been termed ‘Rabak Ware', after a site 70 km to the west on the eastern bank of the White Nile radiocarbon dated by an unassociated shell sample to c. 3200 BC (Haaland 1987: 57). Likewise, similar pots with thick, rolled rims are said to occur at Jebel et Tomat (Clark 1973: 58). Furthermore, Gerharz (1994: 334) claims similarities for this pottery tradition with Kerma, C-Group and Butana Group wares, as do Haaland (1987) and Clark and Stemler (1975), although the Butana comparison has been recently disputed by Winchell (2013). The notional temporal range of this tradition has been founded on two radiocarbon dates (both of 4200 ± 80 cal BP, 2768 ± 109 BC; UCLA-1874D, UCLA-1874E, CalPal 2007) obtained from charcoal not necessarily associated with human activity (Clark and Stemler 1975) and on a later obtained third date on shell from an unspecified context of 3770 uncal. BP (2179 ± 24 cal. BC, (Laboratoire de Science du Climat et de l'Environnement, Gifsur-Yvette, France) on shell from an unspecified context (Babiker 1984).

### Gerharz's Phase III (800-100 BC)

This phase spans the duration of the Napatan state (*c*. 800-300 BC) and the early part of its Meroitic successor. It is said to feature the first appearance of trade items from the north including metals, faience and glass. Gerharz believed that most of the burials from this phase were confined to the eastern half of the site, with habitation continuing in the western portion. This spatial restriction of the burial ground was also said to reflect the emergence of social élites during this time, with only the élite burials containing grave goods. The new pottery styles were said to comprise channelled, painted and ‘stamped’ wares. Gerharz (1994: 331) concluded that Phase III ended in the first century BC based upon the absence of wheel-made Meroitic pottery.

As part of his doctoral research, one of us (Brass) re-examined the extant archival excavation records of Jebel Moya held at the Duckworth Laboratory for the first time since Addison. These records were combined with the laboratory's osteological database to construct a new, updated and expanded Register of Graves for Jebel Moya. The new Register covers 2791 excavated graves with a total of 3192 recorded burials, 3135 of which are human (the remainder include those of livestock). The social aspects of the individual burials and non-mortuary pottery are being considered in terms of both their composition and their spatial and temporal distributions. Together with the Register, this information forms part of a GIS database established to plot the distribution of the graves in order to assist the on-going re-evaluation of the extant artefactual materials and human remains in order to reassess elements of the site's social organisation.

## Reassessment of the British Museum's pottery assemblages

In order to permit informed analysis of social change in the southern Gezira Plain, Jebel Moya needs to be placed in a secure temporal context. The establishment of a secure occupation chronology is central to improving our understanding of the stratigraphic complexity of the site and for decoding intra-site social variation in material culture. This is being accomplished through three separate, yet interlocking strands: reanalysis of the representative pottery sample at the British Museum; optically stimulated luminescence (OSL) dating of six pottery sherds from the British Museum's collection; and stylistic dating of graves with datable artefacts. Attempts at AMS dating of the bone samples curated at the Duckworth Laboratory were unsuccessful due to a lack of collagen. The application of OSL dating at Jebel Moya represents the first direct, absolute dating of any of its features or material attributes.

A thorough reconsideration of the Jebel Moya pottery has been sorely lacking. Previous attempts (Addison 1949; Caneva 1991; Manzo 1995) failed to move beyond vague and unproductive typological groupings. Instead, this study employed an attribute-based approach focusing on the sherds individual parameters including form, fabric, thickness, surface finish, decorative tools and the motor actions employed in executing decorations. Such a system has advantages over Caneva's (1987) more typological classificatory system, widely used in Sudan, which has been criticised for over-reliance on the appearance of motifs rather than the tools which made them (Haour *et al.* 2010: 4–5) and for making inherent assumptions about the ratio of techniques to motifs (Mohammed-Ali and Khabir 2003: 31).

An attribute-based approach breaks down a vessel into its constituent components which can then be compared intra- and inter-site for coherence (Haour *et al*. 2010). Aims have included quantitatively assessing attributes to provide a better view of evolutionary changes, including those marking distinctive disjunctures, thereby providing a better understanding as to which attributes are culturally and temporally sensitive markers (Garcea and Hildebrand 2009). These attributes allow for subsequent sorting to identify trends and generate relevant typologies through the statistical recognition of attribute clusters. Three assemblages have been grouped from the remaining Jebel Moya pottery assemblage at the British Museum, totalling 486 (mostly) rim sherds attributed to different strata at the site. The sherds curated at the Museum of Archaeology and Anthropology, and at the Petrie Museum, fall within the variability of the three designated assemblages.

### Assemblage 1 (Figure 3a–d)

This Late Mesolithic pottery is a rarity with only 13 remaining sherds. The actual ‘Dotted Wavy Line sherds noted and illustrated by Caneva (1991) could not be relocated in the British Museum collection. Decoration on the remaining sherds is stamped and pivoted comb only. The paste predominantly features sand, usually augmented with bone and mica. There is no burnishing.

**Figure 3.**
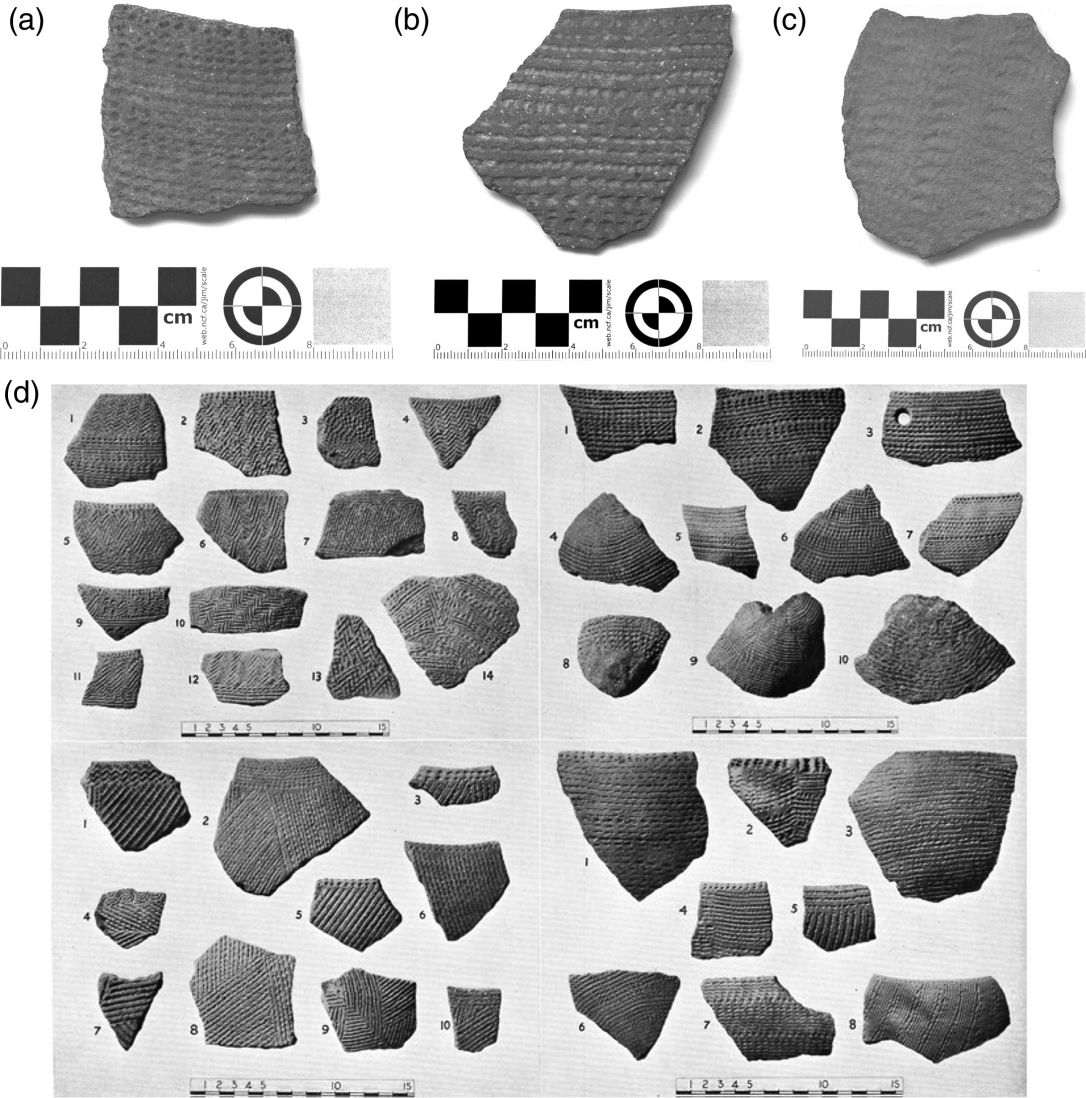
Jebel Moya: Assemblage 1: (a) body sherd 2–3 mm thick with comb-stamped decoration; (b) rim and body sherd 3 mm thick with comb-stamped and pivoted comb décor; (c) body sherd 5–6 mm thick with dragged comb lines and stamped comb décor. The temper of all the sherds is sand paste with bone mica (All from Tray 3. Reproduced with kind permission of the Trustees of the British Museum); (d) a selection of Assemblage 1 body and rim sherds (from Addison 1949: Plate XCIV).

### Assemblage 2 (Figure 4a–d)

These 104 sherds comprise thick (rolled) everted and relatively thinner simple rims with dragged comb and fine spatula-stamped chevrons, as well as fine spatula-stamped impressions on the lip in diagonal or chevron patterns. There are occasional incised fillets. The chevron motif is usually uppermost on vessels, after which there is a band of stamped comb, stylus or impressed cord decoration. Motifs appear on the outer surface of the lips. The temper comprises coarse grit and sand, with mica also sometimes present. Burnishing and slipping occur. The rim angles show predominantly open vessels, with relatively rare closed forms. There are a few sherds combining diagnostic Assemblage 2 features with zoned motifs (in-filled geometric forms) that may date towards the more recent portion of this assemblage; such zoned motifs are common in Assemblage 3.

**Figure 4.**
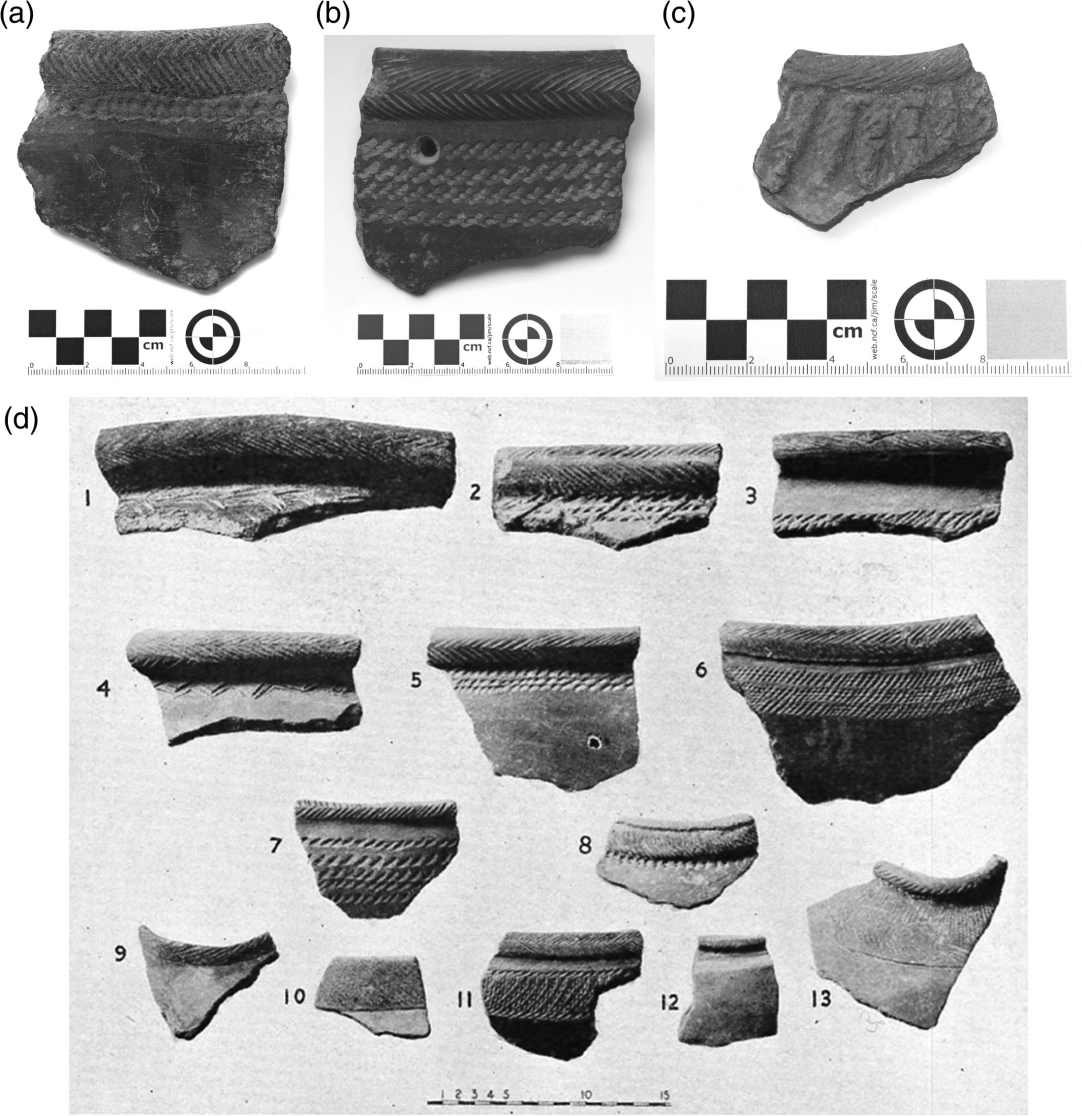
Jebel Moya: Assemblage 2: (a) thick, rolled everted rim and body sherd 5–10 mm thick with dragged comb chevrons on the rim and a comb-stamped line under the lip; (b) thick, rolled everted rim and body sherd 3–24 mm thick with dragged comb chevrons on the lip and a wad of cord impression just under the lip; (c) thick, simple rim and body sherd 8–26 mm thick with incised angular lines on the lip and rows of vertical incised fillets just under it. The temper of all the sherds is coarse grit. (All from Tray 4. Reproduced with kind permission of the Trustees of the British Museum); (d) a selection of large Assemblage 2 rim sherds (from Addison 1949: Plate CIV).

### Assemblage 3 (Figure 5a–d)

This group is distinct from the proceeding assemblages and comprises 369 sherds. Sherds are generally highly burnished, relatively thin (when compared to Assemblage 2) and red slipped. Rim forms are relatively elementary with only simple and everted rims, and most of these are open vessels. Decorative motifs in Assemblage 3 were made using stamped comb, stylus incisions, plain incisions and impressed cord. Also frequently present are pendant triangles (zoned forms) in-filled with fine stamping, either with comb or cord-wrapped elements. There are also occasional examples of cord-wrapped roulettes (impressed, not rolled, *sensu* MacDonald and Manning 2010). Some sherds have motifs on the interior. Critically, most motifs occur on the body of vessels as part of zoned (geometric) forms. Mica temper predominates in the paste with some bone.

**Figure 5.**
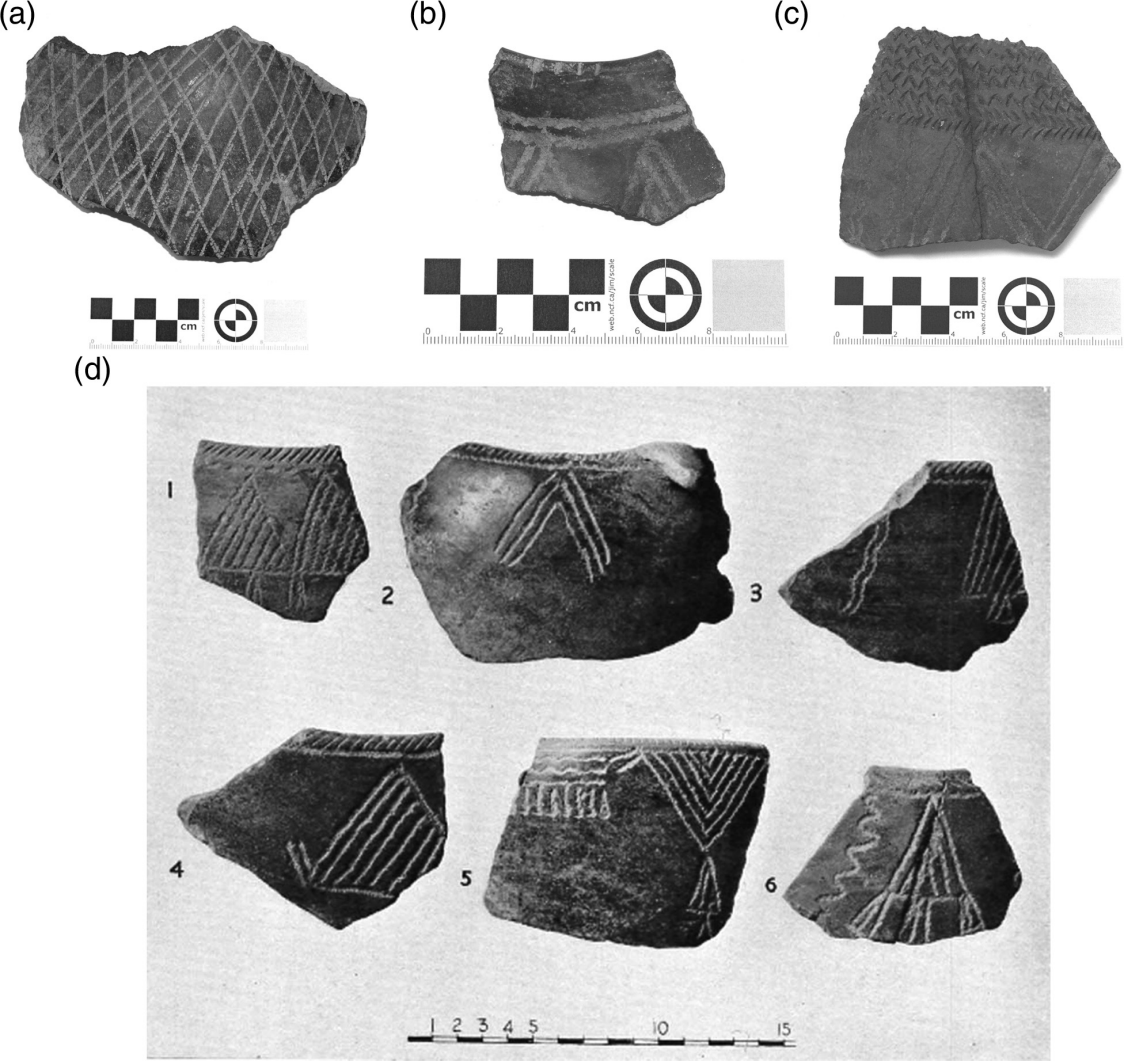
Jebel Moya: Assemblage 3: (a) body sherd 2.5–4.5 mm thick with comb-stamped angular lines forming quadrangles; (b) simple rim and body sherd 3–6 mm thick with two comb-stamped channels under the lip and comb-stamped triangles on the body; (c) body sherd 1–4.5 mm thick with stylus-stamped wavy-lines, stylus-stamped chevron lines and comb-stamped triangular and vertical wavy-lines. The temper of all the sherds is sand with mica (with some organics) (All from Tray 2. Reproduced with kind permission of the Trustees of the British Museum); (d) a selection of large Assemblage 3 rim sherds (from Addison 1949: Plate CI).

**Figure 6.**
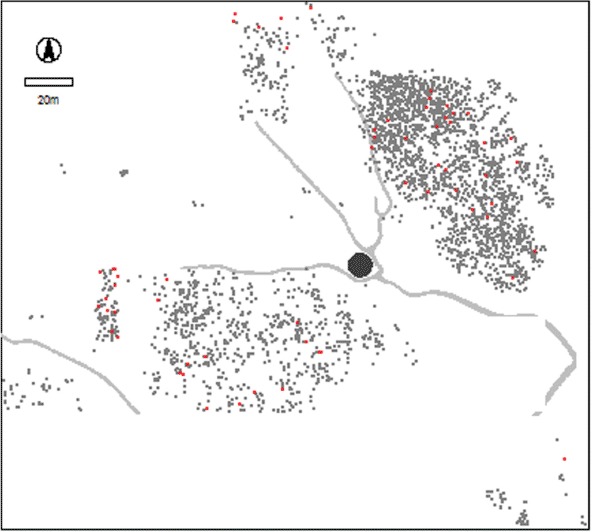
Jebel Moya: the spatial distribution of pottery (red) in recorded association with human burials (grey).

In the new Register of Graves, 77 instances of pottery are recorded in direct association with human burials distributed across the valley (Figure 6, Table 1). Of these 77 burials, 24 (also distributed across the site and through the strata) contain pottery sherds that were illustrated either on the excavation cards or in Addison's publication, or both. Of the 24 illustrated pottery sherds and vessels found in association with burials, only one (Burial 1290) has an Assemblage 2 sherd under its left hand that could have been intrusive. The remainder all belong to Assemblage 3. Furthermore, none of the descriptions of non-illustrated burial assemblage pottery resemble any of the pottery assigned to Assemblage 2; instead, they are all attributable to Assemblage 3.

**Table 1.** Jebel Moya: the breakdown of the spatial distribution of pottery in recorded association with human burials by geographic orientation.

Area	Burials
South	0
Southwest	31
West	0
East	11
Northwest	7
Northeast	23
Unknown	5
Total	77

## The absolute dating of Jebel Moya

Samples from six sherds, three each from assemblages 2 and 3, were prepared for optical dating of coarse-grained quartz (60–125 or 90–255 microns) extracted from specimens supplied by the British Museum. OSL dating should indicate the time that the pottery was fired and has been shown to provide similar accuracy to AMS radiocarbon dating of organic remains in pottery (Manning *et al.* 2011). The samples available for analysis were all pieces measuring approximately 2 × 2 cm and more than 5 mm in thickness that had been removed from a larger original sherd, thus ensuring that a reference specimen was left behind at the museum for future analysis. Sample preparation and luminescence measurements were conducted at the Research Laboratory for Archaeology and the History of Art (Oxford University) using standard preparation procedures (Aitken 1985) that included wet sieving, treatment with hydrochloric and hydrofluoric acids and removal of heavy minerals using sodium polytungstate. All measurements were conducted on an automated Risø luminescence reader using small sized aliquots (2–3 mm) and a single-aliquot regenerative-dose measurement protocol (Murray and Wintle 2000) with the addition of a post-IR blue OSL procedure (Banerjee *et al.* 2001). Luminescence measurements were made at a raised temperature of 125°C, with a preheat 1 (PH_1_) value of 240°C for ten seconds, a preheat 2 (PH_2_) of 200°C for ten seconds and up to six regeneration dose points. Palaeodose estimates were obtained using the weighted mean of between 6 and 12 aliquots derived from an exponential fitting procedure.

Internal and external dose rates were calculated on the basis of geochemical analysis by fusion ICP-MS. No sediment samples associated with the ceramics were available for dose rate determination because the sherds were collected in 1911–1914. However, three of the sherds (X5293, X5294 and X5295) contained small amounts of remnant soil stuck to the surface. This material was considered to be representative of the burial environment at Jebel Moya and was carefully removed and pooled to provide sufficient quantities of material for analysis. An inflated error of 10% was assigned to the external gamma-dose rate (1.03Gy/ka) in order to account for any additional uncertainty on the external dose rate contribution. Radioisotope concentrations were converted to dose rates using the conversion factors of Adamiec and Aitken (1998), the grain-size attenuation factors of Mejdahl (1979) and the absorption coefficient for water by Zimmerman (1971). The contribution of cosmic radiation to the total dose rate was calculated as a function of latitude, altitude, burial depth and average over-burden density based on data given by Prescott and Hutton (1994). It was assumed that overburden accumulated soon after deposition and was negligible relative to the burial period. OSL age estimates were calculated by dividing the mean palaeodose by the dose rate and presented as ± one standard error (Table 2).

**Table 2.** Jebel Moya: summary of the OSL dating results.

Laboratory code	Palaeodose (Gy)	Total dose rate (Gy/ka)	OSL age estimate (years before 2012)
X5291	9.71 ± 1.49	5.52 ± 0.38	1760 ± 295
X5292	16.33 ± 3.64	5.03 ± 0.33	3245 ± 755
X5293	7.19 ± 1.20	4.82 ± 0.32	1490 ± 270
X5294	17.38 ± 2.30	5.06 ± 0.33	3435 ± 260
X5295	17.96 ± 2.14	5.53 ± 0.37	3250 ± 445
X5296	7.58 ± 2.58	4.90 ± 0.33	1545 ± 535

**Figure 7.**
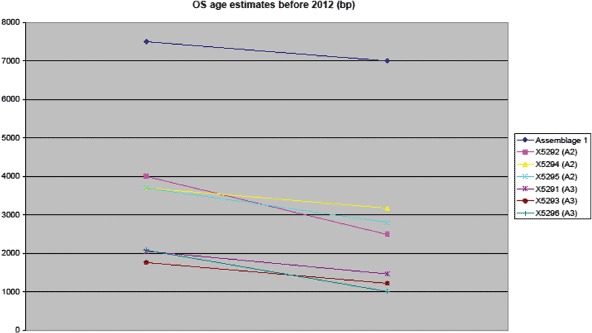
Jebel Moya: the OSL samples from Assemblages 2 and 3, plotted against their date range, showing two distinct clusters. The Assemblage 1 range is hypothetical based on Caneva (1991).

Although the OSL results have very large standard error deviations, there are no overlaps in the respective dates from the Assemblage 2 and Assemblage 3 sherds (Figure 7, Table 3). The results indicate that there were three broad temporal phases. The sherds assigned to Assemblage 1 were not directly dated due to focusing limited dating resources on the more numerous second and third assemblages. The continued relative dating of Assemblage 1 to the sixth or early fifth millennium BC rests on Caneva's (1991) earlier analysis and on subsequent studies of the chronology and distribution of early Sudanese pottery (Jesse 2010; Salvatori *et al.* 2011).

**Table 3.** Jebel Moya: summary of the sampled sherds curated at the British Museum.

Laboratory code	British Museum code	Assemblage	Decorative description	OSL dates
X5291	EA 81191	3	Thin simple rim. Comb-stamped angular pattern on rim. Comb-stamped line on neck. Three comb-stamped triangular-shaped lines on body.	40 BC – AD 550
X5293	EA 81192	3	Thin simple rim. Comb-stamped angular pattern on rim. Cord-impressed line on neck. Cord-impressed and infilled triangles on body.	AD 255–790
X5296	B4 = EA 81191	3	Thin simple rim. Comb-stamped angular pattern on rim. Comb-stamped line on neck.	70 BC – AD 1005
X5292	EA 81192	2	Thickened simple rim. Spatula-stamped pattern on rim with an incised line on neck.	1985-475 BC
X5294	EA 81193	2	Thickened everted rim. Dragged comb chevron-pattern on rim.	1680-1165 BC
X5295	EA 81192	2	Thickened everted rim. Dragged comb chevron-pattern on rim with cord-wrapped impressions below.	1680-790 BC

Assemblage 2 thus comprises occupations covering a relatively long period from the mid-second millennium to the mid-first millennia BC. The nature of the Assemblage 2 occupation at Jebel Moya cannot as yet be determined, but it can be stated with confidence that no burials can be attributed to this period by association with pottery. It thus broadly coincides with the Middle and Classic Kerma Periods in Nubia until shortly after the emergence of the early Napatan élite who ruled in Egypt as the Twenty-Fifth Dynasty *c.* 747-656 BC (Shaw 2000: 482; Hafsaas 2006). No Kerma or Napatan artefacts have been uncovered at Jebel Moya and pottery resembling that from Assemblage 2 has not yet been found further north in the Gezira, although such sherds were found at Rabak to the west and at Jebel et Tomat (of unknown date) to the northwest. Reanalysis and redating of the Rabak assemblages cannot be undertaken due to a lack of uncertainty over where the pottery is located and the site has since been destroyed (Randi Haaland, pers. comm. 2011). The mid-third millennium BC date available for the early occupation of Jebel et Tomat is based on a single radiocarbon date obtained on shell from a soil pit dug on the edge of the midden in uncertain association with cultural materials; Clark (1973: 57) admits that this date needs to be regarded as tenuous. The new OSL dates from the Jebel Moya chevron-decorated ‘thick-wares’ may therefore also have a bearing on the published chronologies of Jebel et Tomat and Rabak.

However, it is noteworthy that the second millennium BC sees increases in ‘thick-wares’ in areas to the east of the Gezira: the ratio of everted thick-wares, some with the distinctive stylus-stamped chevron patterns on the rims, increases in the Late Gash period (*c.* 1700–1500 BC) in the Kassala and wider Gash Delta areas, for example (Figure 1) and is a major presence in its subsequent Jebel Mokram phase (Andrea Manzo and Valentina Perna, pers. comm.).

**Figure 8.**
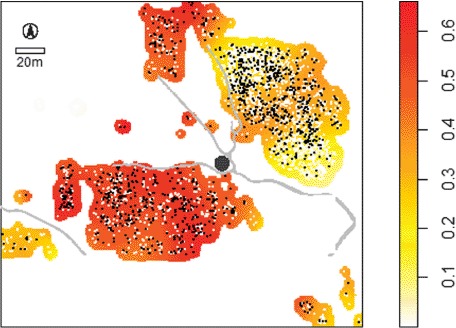
Jebel Moya: the relative density of burials with grave goods to burials without grave goods is greater in the southwest and north (>0.5) than in the east and northeast.

The majority of the extant sherds come from Assemblage 3, which has now been OSL-dated from the first century BC until the mid-first millennium AD. This timespan covers the middle and late Meroitic periods, as well as the aftermath of the breakup of the Meroitic state, which had stretched south into the Butana and with possible settlements along the Blue and White Niles. It is to this phase that the majority of the burials at Jebel Moya may now be assigned, effectively to a society living on the southwestern frontier of the Meroitic kingdom. Of the 3135 human burials, 1108 (35.3%) have associated grave goods, leaving 2026 burials (64.7%) without goods or with artefacts listed as coming from the grave infill. Contrary to the view expressed by Gerharz (1994), GIS analysis reveals that the distribution of grave goods is not concentrated in the east and northeast of the valley (Figure 8). This includes both imported items and items made from imported materials as accompanying burial goods. None of the grave goods (imports) are diagnostic of any temporal period earlier than the late first millennium BC and the only ceramics definitely associated with graves come from Assemblage 3 (see above). This revises the chronological reconstruction of Gerharz (1994: 331), who admitted that his notional end date for the site's sequence of the first century BC was guesswork: the absence of Meroitic pottery from Jebel Moya, and likewise the lack of Jebel Moya Assemblage 3 pottery at Shendi Reach, are not by themselves reliable chronological indicators when the new OSL dates are considered.

Assemblage 3 is contemporary with the later occupational phase of Jebel et Tomat, which five conventional radiocarbon dates place between the early first and the end of the fourth centuries AD (Clark and Stemler 1975). The pottery from this phase at Jebel et Tomat is claimed by Clark 1973 to have strong similarities to Jebel Moya's Assemblage 3, being thin and burnished, although this cannot as yet be verified due to the lack of published illustrations. Clark (1973: 58) also claimed that Jebel Moya and Jebel et Tomat were used by societies ‘sharing common cultural traits.’ As at Jebel Moya, no earthen or stone habitations were evident at Jebel et Tomat. However it is notable that domesticated sorghum was identified at Jebel et Tomat along with the cattle bones, allowing Clark (1973) to propose a model of transhumance between the Nile and the jebel, with dry sorghum cultivation practised in the uplands.

**Figure 9.**
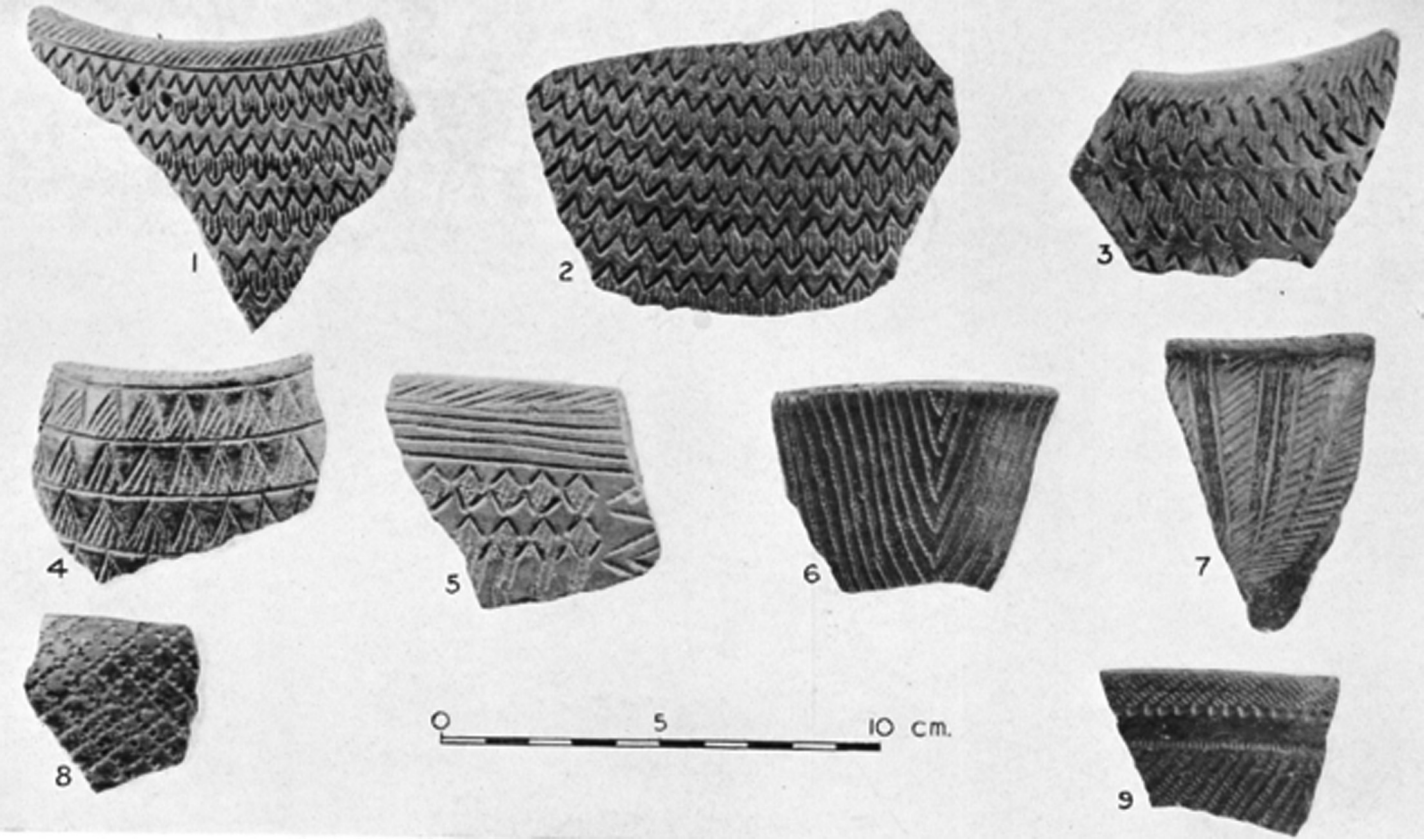
Abu Geili: pottery: 1–3 and 5 stylus-stamped wavy lines. 4 and 6–9 comb-stamped decoration sometimes within incised lines. All are burnished black and brown sherds originally infilled with red pigment (from Crawford and Addison 1951: Plate XXXVIIIB).

**Figure 10.**
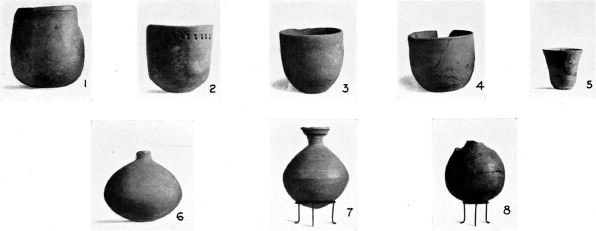
Abu Geili: locally produced wheel-made pottery (from Crawford and Addison 1951: Plate XLIII).

**Figure 11.**
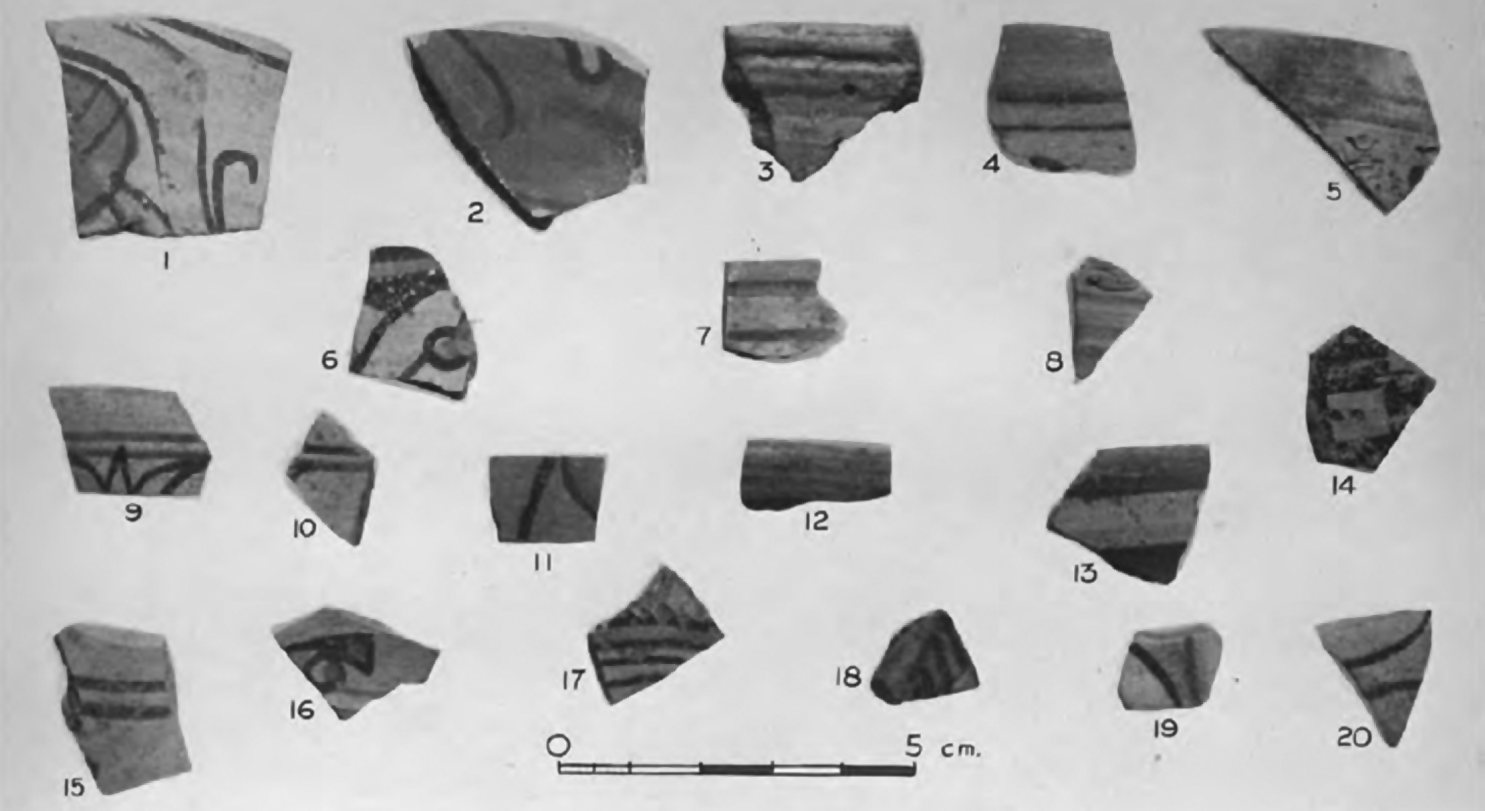
Abu Geili: painted Meroitic pottery (from Crawford and Addison 1951: Plate XLA).

Assemblage 3 is also contemporary with the establishment of a settlement at Sennar, postulated by Addison (1950) to have been a trading station, and of an agro-pastoral settlement at Abu Geili (Addison 1950; Crawford and Addison 1951). Both of these sites are approximately 30 km to the east of Jebel Moya on the banks of the Blue Nile. Assemblage 3 pottery was found at Abu Geili together with locally manufactured wheel-made pottery (Figures 9 and 10) (Crawford and Addison 1951: 44), though not at Sennar, while Meroitic painted pottery was present at both Abu Geili (Figure 11) and Sennar, but not at Jebel Moya. It is during the time of these sites’ notional occupation in the first centuries AD that a southward expansion of Meroe into the western Butana has been postulated (Bradley 1992).

Sites to the south of Jebel Moya, including rescue excavations in that part of the Upper Blue Nile to be flooded by the Rossairis Dam and surface collections from west of the White Nile have yet to be published (Hatim Elnour, pers. comm. 2012). Since these pottery collections have yet to be placed in a secure, radiometrically dated chronological framework potential comparisons with Jebel Moya must remain problematic.

This brief review, emphasises that in the long-term the new OSL dates from Jebel Moya and the formation of a firm pottery assemblage framework open up new opportunities for research in the region by their potential to serve as a reference point against which the artefacts from other sites in the southern Gezira and nearby areas can be potentially dated. They can therefore hopefully serve as a catalyst for further refining localised chronologies.

## Discussion and conclusions

The nature of the populations at Jebel Moya during the time period represented by Assemblage 3 remains unresolved. Rachel Hutton MacDonald (1999) compared samples of teeth from Jebel Moya with those of ethnographically and archaeologically known hunter-gatherer, pastoralist and agriculturalist societies. Dental caries occur when the pH of the oral environment remains consistently below 5.5, causing the dental enamel to become demineralised. In total she examined 2411 teeth from Jebel Moya where the incidence of caries, expressed as a proportion of the total number of teeth examined, was 0.2% (MacDonald 1999: 161), which groups them together with known (modern) pastoral societies. By contrast, the value for samples from Meroitic Nubia (581 teeth) was 15.1% (MacDonald 1999: 161). Furthermore, the Jebel Moya caries occur most frequently on the third molar, whereas caries occurs most frequently on the second molar in the known (semi)-sedentary agricultural populations studied.

It is also worth noting that there are, in total, 55 occurrences of cattle bones among the burial assemblages, either as parts of the animal (e.g. foot) in association with a human burial or as a separate cattle inhumation. Several small clay cattle figurines were also found, though none were part of the burial assemblages. Furthermore, there are no artefacts at Jebel Moya such as sickles or hoes that might indicate harvesting and only one grindstone was found in the burial assemblages. Counterpoised against this information privileging a (specialised?) pastoral economy is the evidence from the contemporary occupation at Jebel et Tomat, where both domesticated sorghum and numerous grindstones occur (Clark 1973; Clark and Stemler 1975). As no botanical analysis was done at Jebel Moya, it is unknown whether domesticated or wild cultivated sorghum was present there, if at all. It may appear then that the southern Gezira Plain was occupied by societies both with a greater and lesser degree of mobility associated with pastoralism, which would mirror the situation in the neighbouring Butana region (Bradley 1992).

The new dates from this study thus provide us with a fresh opportunity to understand part of the archaeological scale and changing nature of interaction in the southern Gezira between notionally stateless populations and Meroitic settlements along the Blue Nile, and to contrast these social dynamics with what was occurring at the same time in the neighbouring Butana and areas farther south. Very little is known about the nature and extent of the Meroitic state's political, ideological and socio-economic reach southwards into the heart of the Gezira Plain (south-central Sudan) and beyond. The trade exchange networks and social organisation of the communities in this region along the southern frontier of the Meroitic kingdom have been little studied apart from some exploratory surveys (Edwards 1989; Fernández *et al.* 2003). Most of the few known sites in the Gezira were found by Sir Henry Wellcome's expedition in 1911–1914, supplemented by subsequent small-scale or brief surveys, particularly in the southern Gezira (Clark and Stemler 1975; Fernández *et al.* 2003). Jebel Moya's geographic location places it in a frontier zone between the states of the Nile Valley (including Meroe) and the relatively little known savanna of modern South Sudan. In light of this, redefining its chronology also alters possible explanations of its *raison d'être.* The first direct dating of selected pottery sherds from different assemblages provides initial temporal ranges for the occupation of Jebel Moya and lays out a chronological backdrop allowing us to place changing social complexity in a broader context.

As discussed above, Gerharz (1994) correctly designated three phases of occupation, but his temporal estimates for the second and third phases were incorrect. Contrary to his interpretation, the apparent absence of Meroitic wheel-made pottery at Jebel Moya is not indicative of the site's abandonment by the end of the first century BC, particularly given the appearance of locally manufactured wheel-made pottery and painted Meroitic pottery at the nearby contemporary Nilotic village of Abu Geili. Rather, we should look to cultural or socio-economic reasons to explain its absence.

Of the three phases of occupation at Jebel Moya, the first conclusive evidence for burial activity comes no earlier than the mid-first century BC. This would appear to indicate that people did not bury their dead where they lived and that there was one primary burial phase that can be best examined as a mortuary complex of a mobile pastoral population contemporary with the Late Meroitic and early Post-Meroitic periods of Nubia. Addison's (19560 reassignment of the cemetery, and Clark's (1973) attribution of its later occupational phase to Meroitic times are thus largely vindicated. Such an occupational sequence of settlements being subsequently used as cemeteries has parallels elsewhere in the Sahelian belt, for example at Dia (Mali) where a first millennium AD cemetery covers a first millennium BC settlement of similar size at Dia Shoma (Bedaux 2005; Bedaux *et al.* 2001).

The temporal continuity of Jebel Moya's Assemblage 3 — from the Late Meroitic into the Post-Meroitic — reorientates the chronological positioning of the burial phase to what may have been the height of the southward expansion of the Meroitic state. The latter's southern frontier is thought to have been in the region of Sennar, to the west of Jebel Moya on the west bank of the Blue Nile (Dixon 1963). Edwards (1999: 91) hypothesised that the cemetery of Jebel Moya may have been the result of communities being forced into the mountain range by raiding conducted by the Meroitic kingdom, or its local élites, into this frontier zone. However, such a model is no longer viable as it would now require the raiding to have continued over the course of up to four centuries and across the time of the breakup of the Meroitic state. Rather, we posit that Phase 3 of Jebel Moya was the mortuary complex of a mobile pastoral community engaged as trade intermediaries with Meroe, passing Sub-Saharan resources northwards via Nilotic trading stations, such as Sennar on the Blue Nile, in exchange for manufactured trade goods (amulets, scarabs, etc.) and non-local raw materials like iron and copper, evidence of which is present amongst the small finds of the cemetery.

Further investigation of social organisation as reflected in the mortuary assemblages of the Jebel Moya, Sennar and Meroitic cemeteries, particularly from the Shendi Reach (Babiker 1985; Edwards 1999), should ultimately shed light on the nature of societies at the periphery of the Meroitic state. Likewise, future work should aim to better illuminate the exchange networks that likely continued to exist as Meroe fragmented in the fourth century AD. The question of why Jebel Moya was abandoned remains unresolved, with one possibility being that the advent of Christianity in the sixth century may have altered pre-existing exchange networks and social relations in the southern Gezira Plain.
